# Data on one-dimensional vertical free swelling potential of soils and related soil properties

**DOI:** 10.1016/j.dib.2021.107608

**Published:** 2021-11-20

**Authors:** Eyo E. U, Uche Onyekpe

**Affiliations:** a Department of Geography and Environmental Management, Faculty of Environment and Technology, Civil Engineering Cluster, University of the West of England, Bristol, United Kingdom; b Department of Data Science, School of Science, Technology and Health, York St. John University, United Kingdom

**Keywords:** Swelling, Soil expansion, One-dimensional swelling, Soil index properties, Moisture content, Surcharge load, Ground movement

## Abstract

Most of the damaging geo-hazards recorded in modern history are caused by soil swelling or expansion. Therefore, proper evaluation of a soil's capacity to swell is very crucial for the achievement of a secure and safe ground for civil infrastructures and related land developments which are founded on the soil. In order to simulate as well as estimate the heave that can occur under field conditions, laboratory one-dimensional oedometer vertical swell-strain testing are most frequently used. Hence, in this brief, one-dimensional swelling tests adopted to measure soil swelling on laboratory-engineered and natural soils covering various regions on the globe are reported. The testing standards and procedures followed in the measurement of one-dimensional swelling are those enumerated in the American Standards for Testing of Materials (ASTM) DDD698, and American Association of State Highways Transport Officials (AASHTO). Slight modifications to the measurement procedures (such as the use of different surcharge loading and custom-made consolidation rings) reflecting special laboratory testing conditions and for the purposes of comparisons, are also reported.

Corresponding soil properties characterising the dataset includes moisture content, void ratio, specific gravity, unit weight, liquid limit, plastic limit, plasticity index, clay content, silt content, maximum dry unit weight, optimum moisture content, and soil activity index, all of which are known to bear either direct or indirect influences on soil. Determination of the state of compaction of the soils where applicable, are carried out based on the American Standards for Testing of Materials (ASTM) DDD698, Turkish Standards (TS), American Association of State Highways Transport Officials (AASHTO)and a combination of both standard and modified efforts. A total of 395 data samples on soil swelling potential are reported. With regards to the corresponding soil properties, a total of 219 data records of soil specific gravity, 321 data records of initial moisture content, 163 data records of void ratio, 273 data records of dry unit weight, 347 data records of liquid limit, 347 data records of plastic limit, 395 data records of plasticity index, 209 data records of activity index, 339 data records of clay content, 174 data records of silt content, 246 data records of optimum moisture content, 228 data records of maximum dry density and 347 data records of Unified Soil Classification System (USCS) are presented. Finally, the dataset of one-dimensional soil swelling described herein are intended to aid geotechnical engineers and researchers who are involved in statistical correlation studies, data analytics, and machine learning predictions using soft computing methods mostly aimed at evaluating soil expansion especially during the preliminary phases of soil investigation and foundation design.

## Specifications Table

**Table:** 

Subject	Civil and Structural Engineering
Specific subject area	Soil swelling and properties of soils.
Type of data	Excel, xlsx
How the data were acquired	Database of soils of low-to-high plasticity subjected to expansion under water were diligently and carefully compiled from literature. Soil swelling data were obtained through laboratory one-dimensional swelling test on soils using the consolidometer (or oedometer) according to standard measurement methods (American Standards for Testing of Materials ASTM D 4546-14 [1], ASTM D 4546-96 [2], American Association of State Highways Transport Officials AASHTO T 258 [3] and slight modifications to these methods. The data on the corresponding index properties of the soils namely moisture content, void ratio, specific gravity, unit weight, liquid limit, plastic limit, plasticity index, clay content, silt content, soil activity and compaction characteristics were also obtained using standard laboratory testing methods. The maximum dry unit weight and optimum moisture content were determined by following the American Standards for Testing of Materials (ASTM D 698-07 [4], (ASTM D 1557-07 [5], ASTM D698-00a [6], Turkish Standards (TS1900) [7], American Association of State Highways Transport Officials AASHTO-T99 [8] and a combination of both standard and modified efforts.
Data format	Raw, Filtered
Description of data collection	Both naturally occurring and laboratory-engineered soils are prepared and swelling test carried out to measure one-dimensional expansion. Undisturbed and disturbed soil materials are carefully inserted into oedometer rings and made to sit in the consolidometer between two porous stones with top and bottom of the porous stones lined with filter papers. Water is then gradually introduced into the oedometer (consolidometer) to inundate the soil samples and allowed to undergo free vertical swelling under a surcharge load for a minimum period of 24 h until equilibrium is attained. On the other hand, standard laboratory testing procedures and indirect calculations are followed to obtain the index properties of the soils as follows: • Liquid limits – using cone penetrometer (fall cone device) methods and a standard Casagrande cup. • Plastic limit – the crumbling of moist soils through repeated remoulding into a small ball and manual rolling into 3mm thread. • Plasticity index – obtained as a difference between liquid limit and plastic limit. • Moisture content – oven drying method • Void ratio – indirect determination by first calculating the volume of soil solids and then subtracting the volume of solids from the total volume to obtain volume of voids as a ratio • Specific gravity of soils – using laboratory volumetric flask to determine the ratio of the weight of a given volume of soil at a specified temperature to the weight of an equal volume of water at the same temperature. • Dry unit weight – indirect determination of the ratio of weight of dry soil to the total volume of the soil. • Activity index – indirect determination by dividing plasticity index by the percentage of clay-sized fractions. • Clay and silt content - standard testing methods for gradation (Particle-Size Distribution, PSD) of soils using sieve analysis. • Optimum moisture content and maximum dry density – soil static and dynamic compaction test methods using standard laboratory moulds with application of standard and modified efforts. • The classification of soils is made according to the Unified Soil Classification System (USCS).
Data source location	Global – Asia, Africa and Europe.
Data accessibility	Repository name: Github.com Data identification number: 2110.04941 Direct URL to data: https://github.com/onyekpeu/Data-on-one-dimensional-vertical-free-swelling-potential-of-soils-and-related-soil-properties-
Related research article	*E.U. Eyo, S.J. Abbey, T.T. Lawrence, F.K. Tetteh, Improved prediction of clay soil expansion using machine learning algorithms and meta-heuristic dichotomous ensemble classifiers, Geosci. Front. 13 (2022) 1–17.*10.1016/j.gsf.2021.101296. [9]

## Value of the Data

• This data is very useful to guide the geotechnical design of foundations of structures that are constructed on expansive soils
• Geotechnical engineers, researchers, and ground engineering experts who are involved with and interested in the accurate recognition and evaluation of expansive soil's capacity to swell during the preliminary stages of soil investigation, site characterisation and general soil expansion and ground movement research, will benefit from this data.
• This dataset can provide insight into the influence of index and intrinsic soil properties on swelling when adopting statistical correlation techniques, data analytics and predictions using soft computing methods.

## 1 Data Description

Dataset of 395 number of soils swelling measurements collected from one-dimensional vertical movement of soils and corresponding soil properties are presented. This dataset references studies spanning over 5 decades leading back to the period when important discourse on soil swelling-related disasters were first considered. The dataset consists of both naturally occurring (disturbed and undisturbed soils) and a mix of laboratory-engineered-soils. Based on the nature and format of reporting from the different sources, the soils are divided into groups with each having properties collated from the same experimental methods. The dataset for each group are saved in individual tabs within the .xlsx file with the tab names (notations) corresponding to the descriptions on Table 1. Table 2 summarizes the soil characteristics measured, indirectly obtained, and perceived to bear possible influences on the swelling behaviour of the soils when inundated with water and consolidated one-dimensionally. A total of 219 data records of soil specific gravity, 321 data records of initial moisture content, 163 data records of void ratio, 273 data records of dry unit weight, 347 data records of liquid limit, 347 data records of plastic limit, 395 data records of plasticity index, 209 data records of activity index, 339 data records of clay content, 174 data records of silt content, 246 data records of optimum moisture content, 228 data records of maximum dry density, 347 data records of Unified Soil Classification System (USCS) observations are contained in the dataset. The soil property attributes presented in Table 1, correspond to the features in the dataset presented at https://github.com/onyekpeu/Data-on-one-dimensional-vertical-free-swelling-potential-of-soils-and-related-soil-properties.

**Table 1:** Description of groups of datasets.

Dataset notation	Soil description	Free swell test method	Surcharge (kPa)	No. of soil properties	Compaction state	Compaction method
DS-1	Low-medium plastic clayey soils	ASTM D 4546-14 [1]	1	13	Compacted	Standard effort (ASTM D 698-07 [4] and modified effort (ASTM D 1557-07 [5]
DS-2	Disturbed clay samples tested under different conditions	ASTM D 4546 [1]	7	13	Compacted	ASTM D698-07[4]
DS-3	Undisturbed clay samples from trial pits and boreholes	Fixed-ring oedometer	7	7	Non-compacted	NA
DS-4	Laboratory engineered clay (mixture of bentonite and kaolinite in various proportions)	ASTM D 4546-96 [2]	1	9	Compacted	Turkish Standards (TS1900) [7]
DS-5	Disturbed expansive clay soil samples	ASTM D4546-14[1]	1	9	Compacted	ASTM D698-00a [6]
DS-6	Disturbed expansive clay soil samples	Custom-made (oedometer cell, with a ring of 50 mm internal diameter and 30 mm height)	0	9	Compacted	Kolay and Singh [10]
DS-7	Disturbed expansive clay soil samples	Standard fixed ring consolidometer using stainless steel rings, 75 mm inside diameter and 19 mm height	7	10	Compacted	Standard Proctor energy: 593.7 kJ/m^3^; Reduced Modified Proctor energy: 1616 kJ/m^3^; Modified Proctor energy: 2693.3 kJ/m^3^[11]
DS-8	Undisturbed expansive clay shales	ASTMD4546-96 [2]	7	7	Non-compacted	NA
DS-9	Disturbed expansive clay soils	Conventional oedometer	6.9	6	Compacted	Standard Proctor
DS-10	Various disturbed soil samples	Custom-made (2 cm high consolidation ring	6.9	5	Compacted	AASHTO [8]
DS-11	Laboratory engineered clay (Silica sand and artificial clays)	Custom-made	6.9	7	Compacted	AASHTO-T99 [8]
DS-12	Laboratory engineered clay (mixture of bentonite and kaolinite in various proportions)	ASTM D4546-96[2]	1.94	8	Compacted	Statically to specified moisture content.
DS-13	Undisturbed expansive soil samples	ASTM D4546 – 96 [2,12]	7	8	Non-compacted	NA

## 2 Experimental Design and Methods

Rigorous systematic literature studies were conducted to obtain the dataset. The literature search included data spanning across research articles and reports with experimental investigations conducted on various soil samples. The methodology of data collection involved thorough screening of articles’ titles, keywords and abstracts that met the criteria of one-dimensional swelling for both compacted and non-compacted soils. Most of the collected data were derived from article tables and where data were only presented on graphs, WebPlotDigitizer [13] was used for data extraction.

Relevant data of soil properties not included in the original data such as the Plasticity Index (PI) and Plastic Limits (PL) of the soil were derived from the parameters that were given as follows:

(1) PI=LL−PL

Or

(2) PL=LL−PI

where LL refers to the liquid limit.

In areas where soil Activity index, (A) was not given, this was calculated as follows:

(3) $$A=\;\frac{PI}{2CF}$$

where CF is the clay-sized fraction.

To allow for an easy and extensive assessment and analysis of the degree of swelling of the different soils and their properties obtained from various sources, entries into each column of the dataset should be manually synchronised to account for missing data through the removal of such affected rows.

**Table 2:** Soil swelling percent and corresponding soil properties.

Soil property	Abbreviation/symbol	Unit
Swell percent	SP	%
Liquid limit	LL	%
Plastic limit	PL	%
Plasticity index	PI	%
Moisture content (initial)	Mc	%
Void ratio	e	-
Specific gravity	G	-
Dry unit weights	γ_d_	kN/m^3^
Activity index	A	-
Clay content	CC	%
Silt content	Sc	%
Optimum moisture content	OMC	%
Maximum dry density	MDD	kN/m^3^
Unified soil classification system	USCS	-

## Ethics Statements

The authors declare that the present work did not include experiments on human subjects and/or animals.

## Funding

This research did not receive any specific grant from funding agencies in the public, commercial, or not-for-profit sectors.

## CRediT authorship contribution statement

**Eyo E. U:** Conceptualization, Methodology, Software, Data curation, Writing – original draft. **Uche Onyekpe:** Visualization, Investigation, Writing – review & editing.

## Declaration of Competing Interest

The authors declare that they have no known competing financial interests or personal relationships that could have appeared to influence the work reported in this paper.
